# Territorial Constraints on Trap–Neuter–Return in Insular Landscapes: Demographic and Ecological Implications of a Conservation-Oriented Policy

**DOI:** 10.3390/ani15243576

**Published:** 2025-12-12

**Authors:** Ruth Manzanares-Fernández, José Martínez-Campo, María del Mar Travieso-Aja, Octavio P. Luzardo

**Affiliations:** 1 Council for Transparency and Good Governance (CTBG), Independent Administrative Authority, Gobernment of Spain, Calle José Abascal, 2, 28003 Madrid, Spain; ruth.manzanares@consejodetransparencia.es; 2 Independent Researcher, Avda, Ciudad de Barcelona, 6, 28007 Madrid, Spain; jose@apamasvida.onmicrosoft.com; 3 Research Institute of Biomedical and Health Sciences (IUIBS), University of Las Palmas de Gran Canaria, Paseo Blas Cabrera “Físico” s/n, 35016 Las Palmas de Gran Canaria, Spain; marimar.travieso@ulpgc.es; 4 Spanish Biomedical Research Center in Physiopathology of Obesity and Nutrition (CIBERObn), Avenida Monforte de Lemos, 5, 28029 Madrid, Spain

**Keywords:** free-roaming cats, sterilization programs, population dynamics, stochastic population modelling, island ecosystems, protected areas management

## Abstract

Managing community cats on islands requires reconciling animal-welfare mandates with biodiversity protection under real operational constraints. In the Canary Islands (Spain), national Law 7/2023 endorses ethical, non-lethal colony management, while subsequent regional resolutions restrict TNR in and around protected areas, narrowing municipal room for action. We combine a multilevel governance assessment with stochastic demographic simulations parameterized from official records to compare three sterilization regimes over 20 years. The intensive regime (≈60–70%/year) reflects the coverage threshold previously identified by Spain-based modelling and field evaluations and adopted in national program guidance; the 20%/year regime represents the pre-resolution baseline widely observed across the archipelago up to December 2024; and the 4%/year regime reflects the post-resolution reality, with abrupt declines in sterilizations, operations largely confined to urban cores, and program suspensions in multiple municipalities. Minimal (4%) and low (20%) efforts produce rapid population growth, bringing numbers close to the assumed carrying capacity under our deliberately high-K configuration and sustaining high densities and associated welfare and ecological risks; only sustained high-coverage TNR prevents saturation and produces progressive declines across island contexts. Under insular constraints, outcomes are determined by achievable coverage rather than regulatory intent; aligning policy and implementation to secure continuous, high-coverage TNR—particularly in risk-sensitive areas with appropriate safeguards—offers a feasible pathway to meet animal-welfare obligations while limiting ecological pressure.

## Simple Summary

In late 2024, a regional resolution restricted Trap–Neuter–Return (TNR) in protected and peri-natural island areas, disrupting municipal programs. We modelled three realistic efforts over 20 years: high coverage (~60–70%/year), the pre-resolution baseline (~20%), and today’s reduced activity (~4%). Only high, territory-wide TNR—continuous and including protected areas—prevents saturation and steadily reduces cat numbers; lower efforts quickly reverse progress and raise risks. Aligning policy with sustained, island-wide TNR offers a practical, welfare-oriented path forward.

## 1. Introduction

The management of community cats in insular territories presents a political, legal, and ecological challenge of growing complexity. Domestic cats (*Felis catus*) are recognised as one of the leading human-associated threats to native biodiversity, particularly on islands where endemic fauna has evolved with limited exposure to mammalian predators [1,2]. At the same time, high-density, unmanaged colonies experience substantial welfare problems linked to disease, conflict, abandonment, and human cruelty [3,4,5,6]. Addressing the issue requires balancing two public policy objectives that, although theoretically compatible, often come into conflict in practice: animal protection and biodiversity conservation This tension is particularly acute in regions of high biological richness, such as the Canary Islands, where the coexistence of feline populations and endemic fauna gives rise to ethical, legal, and governance dilemmas [6]. Even where anthropogenic food subsidies are abundant and colonies are regularly fed, many free-roaming cats retain some hunting behaviour, so human-provided resources can raise demographic carrying capacity and sustain ecological risk unless they are coupled with effective population control [1,2].

Law 7/2023 on the protection of animal rights and welfare marked a paradigm shift in Spanish public policy by establishing the Trap–Neuter–Return (TNR) method as an ethical management tool for feline colonies and by prohibiting lethal population control. The law assigns municipalities operational responsibility under technical supervision to implement non-lethal, transparent management [7,8]. In December 2024, two regional administrative instructions were issued that prohibit TNR inside protected areas and their immediate buffers, restrict trapping and release around Natura 2000 sites and other sensitive habitats, and ban the establishment of new feeding points in these zones, thereby sharply limiting municipal room for manoeuvres in managing community cats where ecological risk is highest. Although intended to prevent negative impacts on native species, these communications—neither processed through public information procedures nor published in any official bulletin—have been treated by local authorities as binding guidance [Documentation available upon request from municipal records and official correspondence archives]. In practice, they substantially limit TNR across extensive parts of the territory, creating direct normative tension with Law 7/2023. According to the definition stated in Law 7/2023, in this paper, we use ‘community cats’ to refer to free-roaming cats that live predominantly outdoors and are not confined to a household, including unowned feral cats, semi-owned cats and loosely owned outdoor cats supported by caregivers, while strictly indoor pets fall outside the scope of our analysis.

In practice, the overlap of mandates between the national Directorate-General for Animal Rights and the regional directorates responsible for natural areas and biodiversity has reduced municipal room for action. The regional bodies do not regulate neutering surgery itself, but they determine where trapping, release and feeding may legally occur; by restricting these activities in and around protected areas, they render TNR-based colony management unfeasible across large portions of municipal territory. As a result, local authorities cannot resort to lethal methods—prohibited nationally—nor apply TNR in restricted zones—limited regionally—thus producing operational gaps that hinder timely, territory-wide interventions [9,10,11]. Given the close interdigitation of protected areas with peri-natural, rural and urban–rural fabrics, restricting TNR in those segments effectively prevents municipality-wide reproductive control, as evidenced by Canary Islands land-use and socio-ecological analyses that map protected cores and buffers alongside adjacent human and tourism–urban landscapes [12,13].

**Figure 1:**
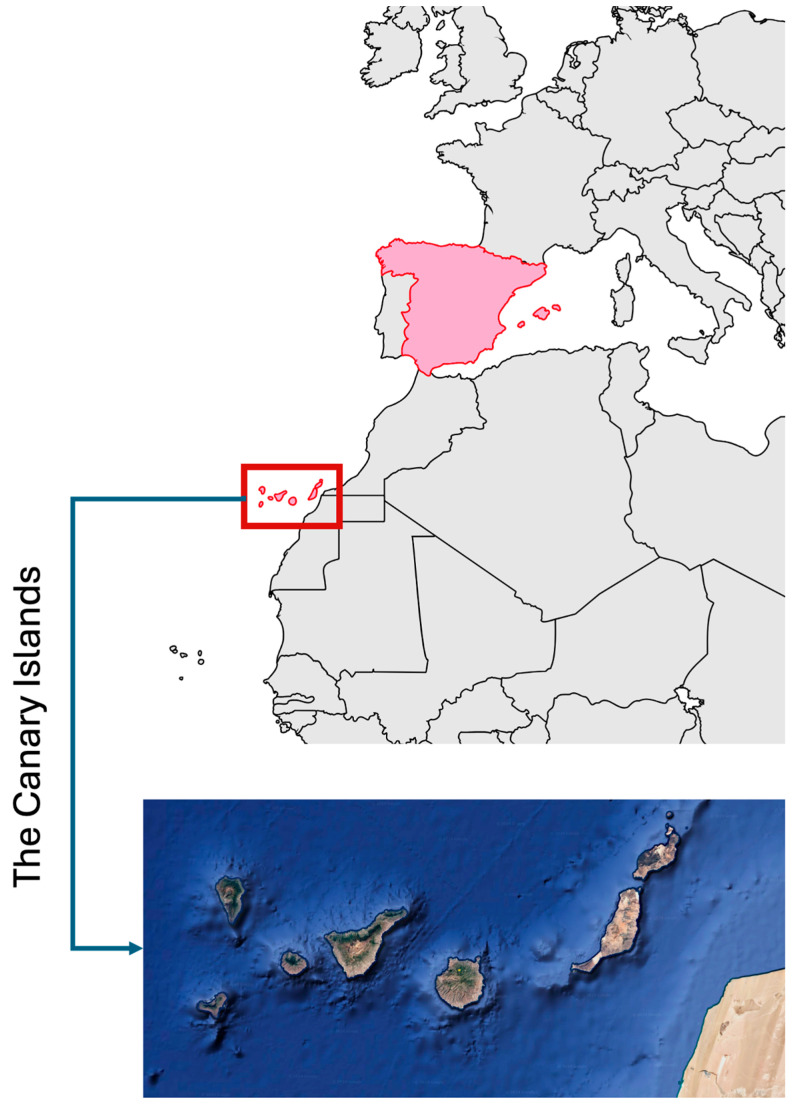
Geographic location of the Canary Islands, belonging to Spain (pink-coloured area). The **upper** panel shows the position of the archipelago with respect to mainland Spain and north-west Africa; the **lower** panel provides a satellite view of the main inhabited islands used in this study. Source: Google Maps (accessed on 13 November 2025).

The Canary Islands are a globally recognised biodiversity hotspot where the tension between free-roaming cats and highly endemic fauna unfolds under a complex, multi-layered governance system (Figure 1).

The archipelago harbours high levels of endemic biodiversity protected under European and national legislation, and a large share of its territory is legally protected, reflecting its biogeographical importance [14]. Land-use analyses show growing urban agglomerations and strong tourism pressure on peripheral belts, evidencing tight interfaces between protected cores/buffers and human landscapes [13]. Socio-ecological work in Fuerteventura further documents the coupling between environmental assets and the tourism system within Biosphere Reserve zoning [12]. In this fragmented island geography, implementing homogeneous policies is challenging and demands spatial and temporal continuity [13]. In practical terms, cat management in the archipelago is shaped by four nested governance levels: European Union biodiversity directives; Spanish national animal-welfare and conservation law and technical guidance (including Law 7/2023 and the Action Plan for Feline Colonies); regional decrees and administrative instructions issued by the Autonomous Community of the Canary Islands; and local ordinances and programme decisions adopted by municipalities and island councils. At the same time, responsibilities are distributed across local, regional, national and EU levels; where common protocols and stable coordination are missing, multi-level governance research for the Canary Islands reports vertical clashes, horizontal inconsistencies and gaps of accountability that hinder coherent, long-term action [6,15].

Within the research literature, multilevel governance highlights how policy outcomes depend on the interplay of competences across supranational, national, regional, and local levels, especially when objectives are cross-cutting and potentially divergent [16,17]. Empirical studies show that weak inter-institutional coordination can neutralize well-intended policy goals even in normatively advanced contexts [16,17]. In the specific case of community cats, international research consistently identifies TNR as an effective, ethical, and socially acceptable strategy when implemented with adequate coverage and continuity [10,11,18,19]. Demographic models and field programs indicate that sustained sterilization can stabilize or reduce populations within multi-year horizons, whereas under-dimensioned or patchy interventions tend to underperform [10,11,20,21].

At the national level, Spain’s Action Plan for Feline Colonies (PACF) translates the mandate of Law 7/2023 for ethical, non-lethal colony management into technical guidance for municipalities and island councils, adopting a TNR operational coverage target of approximately 60–70% of the reproductive population per year to avoid saturation and progressively reduce community-cat numbers when continuity is maintained [22]. This guidance is based on empirical data from registered colonies—most of them small- to medium-sized groups in the low tens of individuals—and defines the 60–70% value at the colony scale, while in practice it is implemented and monitored as island- or municipality-wide effective coverage aggregated across colonies. By contrast, coverage around 20% per year—representative of the average effort documented in the Canary Islands up to December 2024—has been insufficient to prevent saturation, particularly where abandonment and immigration persist. After the regional restrictions, effective coverage in many municipalities approximates ~4% per year, with work largely confined to urban centres and several programs slowed or suspended—an outlook that predicts rapid approach to carrying capacity and sustained high densities if unchanged [6,11,21]. These TNR coverage–response patterns in cat populations, well described in structured population models and long-term TNR simulations, point to a practical question for insular landscapes: what level of sustained sterilization, applied with temporal and spatial continuity across the whole territory—including protected and peri-natural segments under safeguards—is required to avoid saturation and progressively reduce populations?

This study addresses that question by combining a multilevel governance assessment with stochastic demographic modelling for the Canary Islands. Using official programme records as inputs, we simulate long-term population trajectories under three empirically grounded sterilisation regimes over a 20-year horizon: an intensive regime (≈60–70% of the reproductive population per year, corresponding to the operational target adopted in national programme guidance), a moderate regime (≈20%/year, reflecting the pre-resolution baseline widely observed across the archipelago), and a minimal regime (≈4%/year, representing the post-resolution constraints that largely confine TNR to urban cores).

Our central research question is how multilevel coordination constraints shape attainable TNR coverage and, in turn, long-term population outcomes in an insular setting. Specifically, our objectives are (i) to assess coherence and articulation among administrative levels in community-cat management; (ii) to simulate demographic trajectories under three sterilization regimes using VORTEX; (iii) to relate achievable coverage to outcomes for biodiversity and animal welfare; and (iv) to derive coordinated governance options for insular territories. Our contribution is to integrate institutional analysis and stochastic demographic modelling within a single, decision-oriented framework that allows comparative evaluation of policy-relevant coverage regimes.

## 2. Materials and Methods

### 2.1. Study Design and Mixed-Methods Rationale

This study adopts a mixed-methods approach that links institutional governance analysis with stochastic demographic modelling of community-cat populations [23,24,25]. The qualitative component examines coherence of competences and policy implementation across levels, focusing on how overlapping mandates generate operational tension that can restrict municipal room for action [26,27]. Rather than a legal exegesis, it is a decision-oriented assessment of vertical and horizontal coordination capacity and its consequences for program continuity and coverage [25,27]. The quantitative component applies VORTEX v10.6 to simulate population trajectories under alternative sterilisation regimes [23], using parameters previously validated in Spain and adopted in national guidance for feline colony management [11,22]. Integrating both dimensions allows us to connect institutional constraints with measurable demographic outcomes under realistic field and governance constraints [24,27].

### 2.2. Population Model Design

We modelled the archipelago as an insular metapopulation composed of island-level populations of free-roaming community cats, structured operationally as colonies distributed across each island. The annual sterilisation rates considered in the three scenarios (4%, 20% and 60–70%) are expressed as the proportion of reproductive-age individuals within this free-roaming metapopulation, aggregated across colonies at the island scale. Accordingly, these values represent island-wide “effective” coverage rather than spatially homogeneous rates; within-island heterogeneity is expected, with some local areas experiencing much higher or near-zero TNR intensity depending on accessibility and regulatory constraints. Territories where TNR is currently minimal or absent because of regional restrictions are thus represented as non-treated portions of the same island metapopulation, not as separate unmanaged demographic units. For modelling purposes, island-level populations were stratified by territorial context; islands with both urban and rural baselines were represented by two subpopulations (urban and rural), whereas islands with only a rural baseline were represented by a single rural subpopulation (Table 1).

Within each island, we distinguished urban and rural territorial contexts as broad proxies for anthropogenic resource availability and proximity to sensitive habitats, with urban areas characterised by higher levels of human-provided food subsidies (feeding points, refuse, tourism infrastructure) and rural/peri-natural belts combining comparatively lower but still significant anthropogenic resources with closer interfaces to protected habitats, where urban units correspond to the main municipal seats and consolidated built-up/tourism areas defined in official land-use and census datasets, and rural units aggregate the remaining municipal territory (agricultural, dispersed housing and peri-natural zones) [22]. Simulations were run over a 20-year horizon with 1000 stochastic iterations per scenario to characterize trend stability and variability [23].

We evaluated three institutionally grounded sterilization regimes: an intensive regime (≈60–70%/year) aligned with the operational threshold identified by Spain-based modelling and field evaluations and reflected in national guidance [22]; a low regime (≈20%/year) representing the archipelago-wide pre-resolution baseline documented up to December 2024; and a minimal regime (≈4%/year) representing the post-resolution reality of sharply reduced activity with operations largely confined to urban cores and pauses in several municipal programs. All other demographic features (survival, fecundity, abandonment, adoption/removal) were held constant across scenarios and subpopulations to isolate the effect of differences in island-level effective sterilisation coverage.

Carrying capacity (K) was set to four times the island-specific initial population (K = 4 N_0_), serving as a pragmatic upper bound for comparing trajectories across islands and scenarios rather than predicting absolute limits. This pragmatic choice acknowledges the well-known uncertainty and context-dependence of K in ecological models and insular social-ecological systems; accordingly, results should be interpreted as relative differences, not as absolute saturation thresholds [28,29,30]. Initial population sizes by island and territorial context are reported in Table 1 (base year December 2024), compiled from administrative records and harmonized as described below.

### 2.3. Demographic Variables and Parameters

Vital rates and growth parameters (survival, fecundity, age at first reproduction, sex ratio, maximum age) were adopted unchanged from a previously validated, territorially stratified model for Spain that integrated municipal, bioclimatic, and human-density data, and which underpinned the Spanish PACF [22]. Retaining those values ensures methodological continuity between the national model and this insular adaptation and reflects the ecological relevance of near-continuous reproduction and high dependence on anthropogenic resources in comparable Atlantic and Mediterranean settings (Table 2). In VORTEX, age at first reproduction was set to 1 year and maximum reproductive age to 8 years for both sexes, implying a reproductive lifespan of 1–8 years; this annual time-step approximation slightly delays biological onset (which can occur at 5–7 months in free-roaming cats) but is compensated by the extended reproductive span, consistent with colony data and the national PACF model [22]. Adoptions and other removals from colonies were represented as an archipelago-wide “harvest” of 2387 cats per year, plus an additional 1% to account for euthanasia or perioperative death, allocated across islands and urban/rural subpopulations in proportion to their baseline population sizes (Table 2).

### 2.4. Data Sources, Adjustment, and Validation

Administrative program records from the DGDA for 2023–2025 provided the empirical basis for initial population estimates by island and territorial context [22,33]. Because municipal submissions were temporally heterogeneous, we harmonized 2023 and 2024 records to the 2025 base year using a conservative 20% annual sterilization projection in VORTEX, preserving relative differences among municipalities within each island. Where municipal data were missing or incomplete, we applied a small-area/indirect estimation logic, borrowing strength from demographically comparable localities within each island to derive coefficients for controlled intra-island extrapolation, consistent with standard small-area approaches [34]. Given known under-detection in municipal surveys, initial values are treated as lower-bound estimates. Internal consistency was checked by cross-referencing administrative figures with theoretical values from the national model to ensure traceability of each adjustment and coherence of aggregated results [22].

In December 2024, a regional resolution by the Directorate-General for Natural Areas and Biodiversity restricted colony management in protected sites (Canary Protected Areas and Natura 2000) and, crucially, within 1000-m buffers around those areas and around mapped distributions of protected species (including seabird colonies). In practice, island councils and municipalities treated these criteria as binding guidance, which curtailed or paused TNR across extensive municipal polygons intersecting those buffers. Based on municipal reports compiled in 2025, effective sterilization coverage subsequently dropped to approximately 4% archipelago-wide; we therefore define this as the current “minimal-control” scenario used in the simulations.

### 2.5. Assumptions and Limitations

To prioritize decision-oriented comparability, we adopted several simplifying assumptions. We did not include explicit environmental seasonality; in the Canary Islands, climatic variability is low year-round (mild temperatures and limited day-length swings) and food availability for free-roaming cats is largely anthropogenic and relatively stable across seasons, although provision by caregivers is heterogeneous and frequently insufficient on a per-capita basis in high-density colonies, which can increase demographic carrying capacity even if per capita hunting effort is partially reduced, so reproduction tends to be near-continuous and adding seasonal parameters would increase complexity with limited gain at island scale [6]. Inter-island dispersal was set to zero, and intra-island movements between urban and rural contexts were not modelled. Continuity of intensive coverage is treated as an organizational constraint rather than a biological limit. Carrying capacity (K) was specified as a theoretical upper bound rather than empirically estimated for each island. Dependence on municipal administrative records implies potential undercounting and uneven completeness; initial values should therefore be read as lower-bound estimates consistent with the national model underlying the PACF [22]. These choices may affect external validity at fine scales, but they enable transparent ranking of strategies and identification of coverage thresholds that distinguish stabilization/decline from saturation under the current governance configuration.

## 3. Results

The results of the simulations and territorial analysis are presented below, describing initial densities and projected population trajectories for community cats in the Canary Islands.

### 3.1. Initial Density of Cats

**Figure 2:**
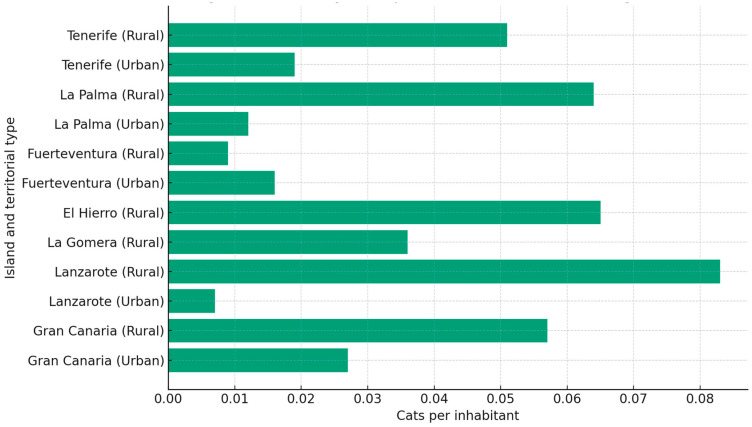
Density of Community Cats per Inhabitant in the Canary Islands (January 2025).

The initial density analysis of community cat populations across the Canary Islands shows a heterogeneous territorial pattern shaped by ecological and demographic factors. Density—reported as cats per inhabitant—is used here as the primary descriptor. Rural-leaning islands present consistently higher cat-to-human ratios than the large metropolitan islands. Lanzarote (0.083), El Hierro (0.065), and La Palma (0.064) lead the distribution—exceeding 60 individuals per 1000 inhabitants—whereas Tenerife and Gran Canaria remain below 30 per 1000, in line with their higher urbanization and waste-management centralization patterns (Figure 2). This gradient is visually apparent, with clusters of high density aligned with small-town and peri-rural mosaics, and lower density pockets around the densest metropolitan corridors. We report these values relative to human population because our primary focus is the management workload and service demand faced by municipalities; when expressed per unit area, the resulting island-level densities are broadly consistent with those described for free-roaming cats in other Mediterranean and temperate urban settings [1,2,4].

When density is considered together with the surface of protected natural spaces, islands such as La Palma and La Gomera emerge as priority territories because they combine comparatively high cat densities with extensive protected land. This co-occurrence does not imply impact per se, but it identifies interfaces where management performance and governance continuity are likely to matter most. In practical terms, these overlays inform the stratified scenario analysis that follows: (i) rural/protected interfaces, (ii) peri-urban belts with comparatively lower cat-to-human ratios, and (iii) dense urban cores with comparatively lower ratios but higher abandonment inflow potential.

### 3.2. Projected Metapopulation Trajectories Under Contrasting Sterilization Regimes

**Figure 3:**
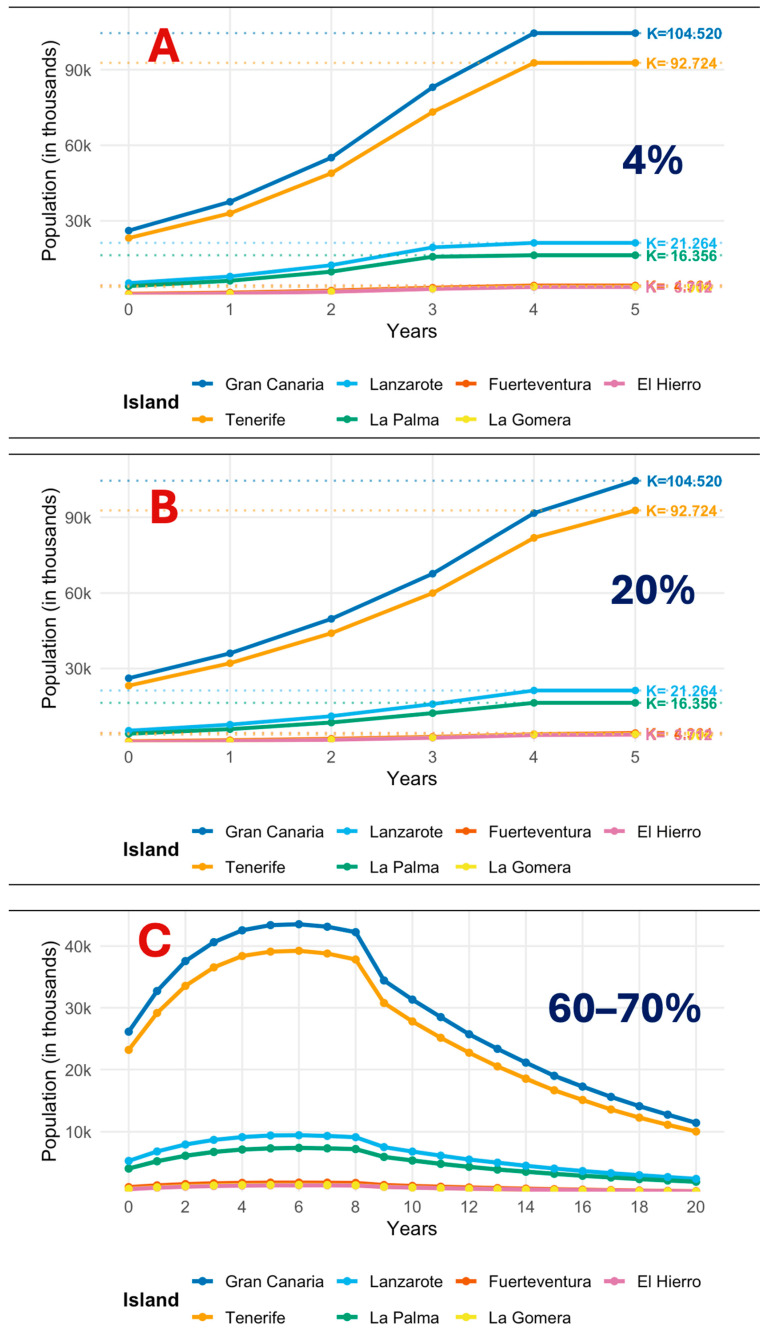
Island-level metapopulation trajectories under contrasting sterilization coverages with high-K assumption. (**A**) 4%: rapid monotonic growth; small islands reach their dotted carrying-capacity lines by years 4–5, while Gran Canaria and Tenerife flatten just below K at year 5. (**B**) 20%: slower growth with visible deceleration after years 3–4; small islands approach K by year 5, whereas the two largest islands remain below K. (**C**) 60–70% (PACF): early crest around years 6–8 followed by sustained decline for the remainder of the 20-year window; no rebounds observed.

Using a deliberately high carrying capacity to isolate the effect of sterilization effort, the three scenarios generate clearly separated trajectories. Under ~4% annual sterilization (Figure 3A), island curves rise rapidly from the initial conditions and bend toward their dotted capacity lines within a few years. Lanzarote and La Palma essentially meet their island-specific K by years 4–5, while Gran Canaria and Tenerife level just below their higher K values (~104.5 k and ~92.7 k, respectively) at the end of the five-year panel. Extrapolated to the 20-year horizon, the archipelago approaches the capacity band late in the window, indicating near-saturation if coverage remains residual.

With ~20% annual sterilization (Figure 3B), growth is slower and visibly decelerates after the mid-horizon point in the five-year panel. Smaller islands approach their K values by years 4–5, but Gran Canaria and Tenerife remain below their capacity lines at year 5. Extrapolated to the 20-year horizon, the archipelago-level trajectory approaches the high-K band, indicating delayed but eventual saturation if coverage does not increase.

Under PACF high coverage (≈60% urban; ≈70% rural) (Figure 3C), all islands exhibit a short initial rise followed by a sustained decline. Inflection occurs around years 6–8 across islands, after which the downtrend is smooth and persistent through year 20. Gran Canaria peaks near ~44–46 k around years 6–7 before contracting to the low-teens by the end; Tenerife peaks slightly earlier and lower (~38–40 k) and trends toward ~10 k at year 20. Lanzarote and La Palma crest near ~9–10 k and ~7–8 k, respectively, and fall to ~2–3 k by the end of the period. No rebounds are observed within the 20-year window.

Table 3 condenses the archipelago-level signal into three numbers: the time to saturation, the year-20 total, and the net change vs. 2025. Under very low (~4%) and low (~20%) sterilization, the system hits the carrying-capacity band within 4–5 years and then remains there; by year 20, both scenarios converge on the same asymptote (≈243,000 cats, a near tripling of the 2025 stock). Only the PACF (60–70%) regime breaks that pattern: it never reaches K and delivers a sustained decline, leaving ≈27,100 cats at year 20—~56% below the 2025 baseline. In short, the table shows that, under a high-K assumption, modest sterilization merely delays saturation, whereas PACF reverses growth and achieves large, system-wide reductions.

### 3.3. One-Year Response (2025) Under Residual Coverage (~4%)

**Figure 4:**
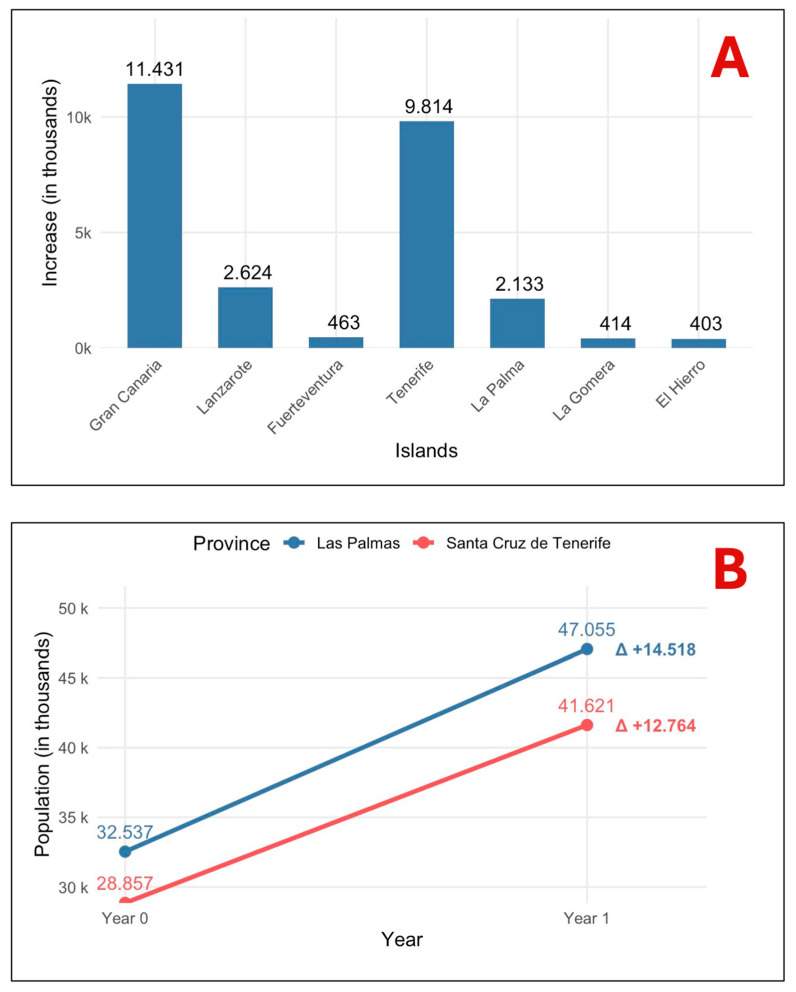
One-year response under residual sterilization (~4%). (**A**) Island-level net increases in 2025; Gran Canaria and Tenerife account for ~78% of the archipelago-wide gain. (**B**) Provincial aggregates show parallel growth (Las Palmas: +14.518 k; Santa Cruz de Tenerife: +12.764 k), with no compensatory declines. Overall, the metapopulation grows by +27.282 k (~+44.4% over baseline) within a single year.

Figure 4 captures what has already occurred in 2025 under ~4% sterilization. Archipelago-wide, the metapopulation grew by ~27.3 thousand cats in a single year—an increase of ~44.4% over the year-0 baseline. The surge is system-wide: Gran Canaria +11.431 k and Tenerife +9.814 k account for ~78% of the total gain, while Lanzarote +2.6 k, La Palma +2.1 k, Fuerteventura +0.4 k, La Gomera +0.4 k and El Hierro +0.4 k also rise (Figure 4A). Aggregated by province, Las Palmas grows from 32.5 k to 47.1 k (Δ +14.5 k; +44.6%) and Santa Cruz de Tenerife from 28.8 k to 41.6 k (Δ +12.7 k; +44.2%), with no compensatory declines (Figure 4B). In practical terms, this first-year uptick establishes a higher reproductive baseline from which subsequent years of the ~4% scenario evolve in the long-term projections.

## 4. Discussion

Coverage around 4 and 20% yields a similar pattern of near-saturation, with trajectories lying within the high-K band for most of the 20-year window, which indicates delayed but not avoided saturation. PACF-aligned coverage near 60–70% follows a different path, with an early crest around years six to eight and a sustained decline that ends near 27.1 thousand cats at year twenty, a reduction of 55.9% relative to 2025. The first operational year under 4% has already added about 27.3 thousand cats across the archipelago, with positive increments on every island. Taken together, these values show that modest effort leads to near-term saturation, whereas high and contiguous effort reverses growth at the metapopulation scale (Figure 2 and Figure 3, Table 3).

These demographic contrasts mirror differences in institutional capacity, coordination across administrations and regulatory coherence. Spain’s decentralized system assigns primary operational responsibility for colony management to municipalities and island councils, while national and regional authorities set the legal framework. In such arrangements, effectiveness depends less on sophisticated legal drafting and more on sustained, coordinated implementation with shared protocols, interoperable data and measurable targets. In the Canary Islands, TNR intensity and coverage therefore vary widely among islands and municipalities, and the post-resolution ~4% regime is not a deliberate policy objective, but the empirical outcome of fragmented, weakly coordinated management combined with spatial restrictions that confine work to urban cores. Under these conditions, two legitimate aims—humane colony management and biodiversity conservation—end up restricting each other in practice: low, uneven coverage predictably drives populations towards saturation, while only high-coverage, territory-wide programmes such as the PACF-aligned scenario are compatible with the stated goals of protecting cats and limiting ecological pressure.

We develop this interpretation in four strands that follow. First comes an institutional and legal reading of the conflict, then the ecological and animal-welfare implications of the modelled trajectories, followed by consequences for multilevel governance and policy design, and finally the social dimension of legitimacy, after which we present limitations and a forward-looking agenda.

### 4.1. Institutional and Legal Interpretation

Community-cat management in the Canary Islands reveals a structural governance conflict within Spain’s decentralized state [15,17]. This is not a mere legal disagreement but a systemic disarticulation among levels of government that share competences yet lack effective mechanisms of collaboration and institutional loyalty [16,27]. At the national level, Law 7/2023 establishes a mandatory system for humane colony management—prohibiting lethal control and assigning municipalities direct responsibility under veterinary supervision [7,8]. In December 2024, however, regional instructions in the Canary Islands restricted TNR in protected natural areas (including peripheral buffers) and within the Natura 2000 Network. The resulting contradiction between the nationally mandated framework for animal protection and the conservation objectives pursued through regional instructions—some of them issued as internal communications without standard publication or public-information procedures—exposes the absence of a cooperative architecture capable of reconciling concurrent objectives within a single governance system [15,27]. Under Law 7/2023 and the national Action Plan for Feline Colonies, the explicit goal of managing free-roaming cats is to reduce the density of outdoor, non-confined cats humanely through high-coverage TNR, thereby improving colony welfare while limiting predation and other risks to biodiversity [7,8,22]. By prohibiting trapping, neutering, return and feeding in protected areas and in extensive buffer zones around them, the regional directorate prevents municipalities from applying that strategy precisely where ecological sensitivity is highest and, as reflected in the ~4% scenario, drives island-wide coverage down to levels that increase rather than reduce predicted ecological pressure [6,11,21].

This tension is amplified by insufficient inter-administrative loyalty and coordination. Municipalities, legally competent in animal protection, are required to implement the national mandate but are materially prevented from doing so in parts of their territory by regional restrictions [7]. The Autonomous Community acts from a conservationist logic without offering operational alternatives responsive to local needs, producing guidance that effectively undermines baseline national law [14]. In this context, fragmented competences and the absence of coordinating leadership transform decentralization into an operational obstacle and a source of paralysis in public-policy implementation [25,26].

The political dimension is also relevant. The partisan distribution of power across national, regional, and island governments has hindered the creation of stable mechanisms for collaboration [24]. Community-cat management has become a symbolic arena where broader ideological disputes over animal protection, conservation, and state intervention play out [24]. In the Canary Islands, weak cooperative architecture, fragmented planning, overlapping competences, and limited participation and data have been documented in other policy arenas, undermining implementation capacity and public trust—patterns consistent with our findings here [35]. This politicization narrows the space for technical cooperation and corrodes institutional trust among levels of government [27].

From a technical-administrative perspective, the problem manifests as a lack of common instruments for planning, evaluation, and accountability. There are no standardized protocols for TNR in protected environments, no shared indicator system to evaluate ecological and welfare outcomes, and no interoperable registry to trace interventions, despite the requirements following Law 7/2023 [7,8]. In the absence of a data-driven, jointly evaluated governance framework, each administration acts according to its own resources and priorities, producing a mosaic of uncoordinated local practices and reinforcing territorial inequality [15,27].

The demographic outcomes of the simulations empirically reflect this institutional deficit. Under low (20%) and minimal (4%) sterilization, populations reach carrying capacity between years four and five—a pattern consistent with the consequences of competential blockage: chronic overpopulation and sustained high densities [9,21]. Conversely, the PACF-aligned scenario (60–70%)—a coordinated, sustained strategy—stabilizes populations and drives progressive decline [10,11,20,36]. From the standpoint of governance theory, this contrast illustrates the gap between institutional design and operational capacity: progressive legal frameworks do not guarantee results unless accompanied by mechanisms for coordination, learning, and adaptation [17,25]. As classic governance scholarship notes, effectiveness in multilevel systems depends less on hierarchy than on the quality of interactions among actors [26,27].

Finally, incoherence between animal-protection and conservation policies triggers a crisis of institutional legitimacy. Citizens perceive the contradiction between advanced normative discourse and visible realities of neglect and ecological deterioration [14]. This misalignment erodes public trust and weakens the constitutional principle of administrative effectiveness [24,26]. The challenge is not recentralization but rebuilding inter-administrative trust and establishing a cooperative governance system capable of aligning public objectives around a shared vision of sustainability, protection, and responsibility [15,27].

### 4.2. Ecological and Animal-Welfare Implications

From a management standpoint, the primary reason to intervene in free-roaming cat populations is twofold: to reduce preventable harm to cats themselves and to limit their cumulative impact on vulnerable wildlife. From a population-ecology perspective, the simulations delineate three trajectories with direct implications for insular biodiversity and colony management. In Scenarios A (4%) and B (20%), the early approach to the high-K band—between years four and five—is consistent with saturation dynamics observed in demographic models of free-roaming cats under low sterilization effort [9,21]. High densities increase the likelihood of negative interactions with native fauna—especially ground-nesting birds, endemic reptiles, and small threatened mammals—while amplifying socio-environmental conflicts linked to unmanaged colonies such as noise, refuse, and vectors [3,4,6]. Saturation can also facilitate pathogen transmission and intensify pressures in sensitive habitats, particularly where colonies abut protected areas or tourism–urban interfaces [37].

From an animal-welfare perspective, persistent high densities degrade living conditions through resource competition, stress, trauma, suboptimal nutrition—including malnutrition in subordinate or sick individuals—and infectious disease when sterilization and veterinary oversight are discontinuous, a scenario that conflicts with the standards and supervisory logic established in Spain’s Law 7/2023 and the national technical guidance [7,8]. In contrast, Scenario C (60–70%) yields a demographic equilibrium compatible with biodiversity-conservation requirements and animal-protection standards: the modeled 55.9% reduction over twenty years lowers potential predation pressure and improves colony health profiles, aligning with empirical reports that sustained, contiguous TNR is required to avoid compensatory effects and secure long-term declines [10,11,19].

Stabilization at scale also improves the efficiency of public and volunteer efforts and facilitates veterinary and behavioural monitoring, which can reduce disease transmission and abandonment [18,38,39,40,41]. Taken together, these results underscore that conservation and animal protection are complementary when management is ethical, data-informed, and coordinated: properly implemented TNR contributes simultaneously to ecosystem protection and the reduction of suffering, whereas absolute prohibitions or administrative inaction tend to produce the very saturation that undermines both aims [3,4,8,42]. Although lethal eradication campaigns have been used in some insular conservation programmes, they conflict with the legal status of cats as protected companion animals under Spanish law and with strong social attachment to community cats in densely inhabited islands; in this context, high-coverage, non-lethal TNR is the only feasible pathway to pursue demographic and ecological goals within an acceptable welfare and policy framework.

In practical terms, effective implementation requires alignment across three planes. Ecologically, risk assessment must be tailored to habitat and island context to prioritize sensitive areas without collapsing territorial continuity [12]. Sanitarily, veterinary registries and epidemiological surveillance should accompany fieldwork to document health indicators and zoonotic risk [43,44]. Administratively, stable funding, inter-institutional cooperation, and unified protocols are necessary to maintain the temporal and spatial continuity that the models and field evaluations identify as decisive thresholds for stabilization and decline [8,11,27].

### 4.3. Implications for Multilevel Governance and Policy Design

The regulatory conflict in the Canary Islands is symptomatic of a structural dysfunction within multilevel governance that may recur elsewhere. The case shows that advanced legislation and technically sound strategies are insufficient when coordination, communication, and evaluation mechanisms are missing, or when policy becomes a political battlefield [16,17]. In the absence of a cohesive cooperative architecture, shared competences drift into vacuums of responsibility, administrative fragmentation, and operational inefficiency that undermine both animal protection and biodiversity conservation [14,15].

International experience—particularly the Policy Coherence for Sustainable Development approach—emphasizes that environmental policy effectiveness depends less on legal drafting than on aligning goals, means, and decision levels within a common accountability framework [27]. In the Canarian context, the lack of such a system has opened three interlocking gaps. The first is a vertical gap between the State, the Autonomous Community, and municipalities, where legal mandates [7,8] are not translated into workable protocols. The second is a horizontal gap among administrations at the same level, where political or ideological differences obstruct technical cooperation [15,16]. The third is a transversal gap separating public institutions from the scientific community and civil society, limiting the incorporation of evidence and stakeholder participation into decisions [17,45].

Bridging these gaps requires an institutional redesign oriented toward a stable cooperative architecture [15,27]. In practical terms, this means establishing permanent technical commissions that bring together state, regional, island, and municipal authorities with scientific and animal-protection stakeholders to validate population-control plans and authorize actions in sensitive environments. It also entails developing regional protocols for ethical colony management in protected areas that are explicitly risk-based—integrating habitat sensitivity, seasonality, colony location and size, and species vulnerability—and that enable conditional authorizations with biosecurity measures, monitoring, and post-intervention audit rather than blanket prohibitions [8,12,46]; deploying a unified, interoperable, and auditable system for data and monitoring to evaluate compliance with Law 7/2023 and the PACF [7,27]; harmonizing local regulation through model municipal ordinances aligned with national standards [8]; and conducting periodic, public ex post evaluations that assess both ecological and welfare outcomes [27]. Achieving coherence across levels of government and ensuring transparent, measurable results are essential to rebuild public trust and to consolidate a governance model grounded in scientific evidence and public ethics [15,17].

### 4.4. Social Dimension: Normative Incoherence and Institutional Legitimacy

Normative incoherence—misalignment among legal frameworks that pursue complementary goals without coordination—constitutes not only a technical shortcoming but a social fracture that undermines trust [27]. In decentralized systems, divergence between national and regional regulations catalyses perceptions of institutional failure, especially in morally sensitive fields such as animal protection and conservation [14,15].

The theoretical premise is straightforward: legitimacy is the social currency of governance. When governmental action appears fragmented or contradictory, citizens infer incompetence or a lack of moral commitment [24,25,26]. Thus, the legitimacy of animal-protection policy depends less on progressive laws than on the capacity of institutions to act coherently across territorial and sectoral boundaries [15,27].

The Canary Islands vividly illustrate this dynamic. The coexistence of a national mandate for non-lethal management [7] and regional restrictions on TNR in protected areas and buffers has created what governance scholarship terms a pathological overlap of competences: two frameworks legitimate in isolation but mutually disabling in practice [16]. For local authorities—obligated to implement national law yet constrained by regional prohibitions—the outcome is paralysis; for the public, the result is perceived arbitrariness, opacity, and institutional neglect [14,27,35].

Empirical patterns reinforce this sociopolitical reading. Under low or absent sterilization, populations reach carrying capacity within a few years, producing visible overpopulation in urban and rural settings [6,9,10,21,40]. Citizens experience the tangible consequences of incoherence: animal suffering, ecological degradation, and the perception of ineffective state control [12,27]. The frustration of animal-protection organizations—seeing progress reversed by political inaction—adds to this climate [6,20]. As classic analyses of political systems note, legitimacy hinges on congruence between normative expectations and observable outcomes; when laws promise humane, science-based management but reality reflects disorder, trust collapses [24,26].

This erosion of legitimacy has recursive effects: as trust declines, civic cooperation and compliance weaken, reducing the effectiveness of subsequent interventions and reinforcing cynicism [17,27]. Within animal protection, this carries an ethical dimension: the State is perceived as failing in its duty toward sentient beings, amplifying the symbolic cost of inaction [15,24,25]. Contemporary society’s rising sensitivity to both animal suffering and biodiversity loss elevates the demand for coherence: citizens no longer accept institutions that invoke conservation and protection while allowing both to be undermined through contradiction or inaction [12,14].

Restoring legitimacy therefore requires institutional reforms that reconnect normative, operational, and perceptual dimensions. Cooperative architectures—permanent intergovernmental commissions, unified data systems, adaptive protocols—must align regulatory objectives with visible, measurable outcomes [15,27]. Only then can the State recover credibility and fulfil its dual mandate: to protect biodiversity and uphold the moral community that binds humans and animals [7,8].

Translating the above into practice requires moving from a compartmentalized scheme of authority to an integrated form of governance guided by three principles. The first is institutional coherence—no regulation or administrative decision should obstruct the fulfilment of another of equal or higher rank—which in this case calls for regional protocols that allow TNR in protected areas when risk assessments so indicate, rather than broad prohibitions [8,27]. The second is shared public responsibility, whereby each level of government contributes resources, data, and evaluation capacity through permanent coordination structures, technical training, and financial cooperation [17,27]. The third is democratic legitimacy, grounded not only in legality but in the ability to meet social expectations for animal protection and biodiversity conservation through coherent, transparent, and measurable action [15,27].

Operationalizing these principles involves aligning ends and means so that conservation and animal-protection goals are matched with functional instruments; generic bans in protected areas, if not paired with risk-based alternatives, impede compliance with Law 7/2023 [7,8]. It also entails replacing all-or-nothing rules with risk-differentiated protocols that integrate habitat characteristics, seasonality, colony proximity, and species sensitivity, enabling conditional interventions supported by biosecurity, monitoring, and post-intervention evaluation [12,46]. A cooperative architecture should be established through permanent intergovernmental technical commissions at regional, island, and municipal levels, working with scientific and animal-protection stakeholders to validate plans, authorize actions in sensitive environments, share interoperable datasets, and audit outcomes [15,27]. Common standards and open data are needed to create a unified, auditable registry of colonies, interventions, and wildlife interactions that strengthens transparency, accountability, and institutional learning [8,27]. Programs should adopt adaptive-management logic with explicit, quantifiable goals at the island scale, predefined decision thresholds to adjust intensity, and periodic public evaluations [27]. Local regulation can be harmonized via model municipal ordinances aligned with PACF and Law 7/2023 [7,22]. Finally, public communication must explain the connection between animal protection and conservation and clarify that the 60–70% sterilization threshold is not an arbitrary figure but an operational benchmark that models and field evaluations consistently associate with stabilization and long-term decline of free-roaming cat populations when applied with sufficient temporal and spatial continuity [8,10,11].

### 4.5. Limitations and Future Research

This study’s limitations are both methodological and institutional. First, analysis depends on administrative records (DGDA, 2023–2025) that are incomplete, temporally inconsistent, and geographically uneven. We harmonized earlier records to the 2025 base year using a conservative 20% sterilization projection, but temporal uncertainty remains and initial values should be considered lower-bound estimates. This reflects a structural issue: the absence of a unified, standardized database for colonies, sterilizations, and trends.

Second, municipal censuses underestimate real populations (commonly 20–30%). No uniform correction factor was applied, to avoid introducing bias given methodological heterogeneity across municipalities. Third, carrying capacity (K) values are theoretical approximations derived from initial totals; while enabling comparability, they likely overestimate environmental elasticity and underrepresent localized saturation near biodiversity hotspots. Fourth, demographic parameters were adopted from a validated national model without local calibration; while ecologically reasonable for Atlantic/Mediterranean islands, future work should refine survival and fecundity using longitudinal field data from Canarian TNR programs and veterinary registries. Fifth, the model assumes no inter-island dispersal and omits intra-island movements between rural and urban contexts, simplifying metapopulation structure and excluding human-mediated movement (relocation, feeding practices).

Despite these constraints, the model yields robust comparative trends sufficient to evaluate the relative impact of sterilization effort on stabilization and to infer institutional implications. The limitations themselves underscore a governance insight: data quality mirrors coordination quality. Fragmented information is not merely a technical problem; it signals systemic disarticulation between administrative levels and policy domains.

Future research should therefore pursue a dual agenda—scientific refinement and institutional innovation. On the scientific side: (i) estimate colony-specific K via GIS, remote sensing, and ecological audits; (ii) develop spatially explicit metapopulation models incorporating connectivity, habitat typologies, and epidemiological dynamics; and (iii) assess the cost-effectiveness and ecological outcomes of varying sterilization intensities, including mixed strategies (TNR, targeted relocation, environmental education). On the institutional side: pilot programs with regional and municipal partners should (i) test risk-based TNR protocols in protected areas, (ii) implement standardized monitoring for colony management and wildlife interaction, (iii) establish intergovernmental technical commissions that integrate scientific data into decisions, and (iv) incorporate ex post evaluation to measure ecological impact and welfare gains. Advancing this agenda will enhance modelling precision and strengthen institutional capacity for evidence-based governance.

## 5. Conclusions

In insular landscapes such as the Canary Islands, long-term population outcomes for free-roaming cats depend primarily on the level of sterilisation coverage that can actually be achieved and sustained in practice, rather than on how ambitious regulatory texts may be on paper. Across a 20-year horizon, minimal and low efforts—approximately 4% and 20% per year—drive populations rapidly towards the high-K band and maintain high densities, whereas only sustained high-coverage TNR (about 60–70% per year), applied with temporal and spatial continuity, prevents saturation and produces progressive declines across island contexts. Because protected natural areas in the archipelago are tightly interwoven with peri-natural, rural, and urban settings, restricting TNR in those segments effectively constrains municipality-wide control and undermines continuity.

These findings indicate that ethical management compatible with biodiversity objectives requires territory-wide, high-coverage sterilization sustained over time, including in risk-sensitive areas under appropriate safeguards. The core implication is operational: aligning governance and implementation to secure continuous, island-scale TNR is a necessary condition to meet animal-welfare obligations while limiting ecological pressure in insular systems.

## Figures and Tables

**Table 1:** Initial population of free-roaming cats in the Canary Islands by territorial type (base year December 2024). Source: authors’ elaboration from General Directorate of Animal Rights (DGDA, Government of Spain) grant data (2023–2025).

Island	Territorial Type	Human Population	Initial Cat Population
Gran Canaria	Urban	765,365	20,483
Gran Canaria	Rural	98,578	5646
Lanzarote	Urban	113,479	770
Lanzarote	Rural	54,547	4546
La Gomera	Rural	22,436	813
El Hierro	Rural	11,806	773
Fuerteventura	Urban	51,307	826
Fuerteventura	Rural	31,098	265
La Palma	Urban	20,283	250
La Palma	Rural	60,262	3838
Tenerife	Urban	867,377	16,386
Tenerife	Rural	132,324	6795
**Total**	**—**	**2,228,862**	**61,392**

**Table 2:** Key demographic parameters and assumptions used in the Vortex population model for community cats in The Canary Islands.

Parameter	Value/Assumption	Justification
**Carrying Capacity (K)**	4× initial population	Reflects ecological resilience and prevents unrealistic exponential growth.
**Reproductive Rate**	Up to 3 litters/year per breeding female	Based on biological potential, adjusted by Reproductive Utilization Rate (RUR) [22].
**Litter Size (mean ± SD)**	Urban: 3.75 ± 1.2 kittens; Rural: 4.75 ± 1.3 kittens	Derived from field studies and literature on urban vs. rural populations.
**Kitten Mortality (<1 year)**	65%	Conservative estimate based on unmanaged colony [31,32].
**Adult Mortality (>1 year)**	15%	Standardized across scenarios for comparability; aligns with feral cat studies.
**Reproductive Lifespan (both sexes)**	1 to 8 years	Conservatively set due to Vortex’s annual timestep limitations; avoids underestimation of long-term reproductive contribution. Minimum breeding age 1 year; maximum 8 years, reflecting annual time step and field-based reproductive span.
**Sterilization Impact**	100% reproductive suppression in sterilized individuals	Modelled via Vortex “dispersal” function to dynamically transfer to sterile pool.
**Sex Ratio at Birth**	50% males: 50% females	Standard assumption in cat demographic models; consistent with national PACF model.
**Disease Outbreaks**	5% annual probability; 30% increased mortality if triggered	Model stochastic catastrophic events (e.g., panleukopenia, poisoning).
**Abandonment Rate**	1240 cats/year, distributed across 8 scenarios (RL, RM, RH, etc.)	Based on 2024 national survey of 500 municipalities (DGDA data); proportional allocation by scenario.
**Adoption and Removal Rate (“Harvest”)**	2387 cats/year (archipelago-wide total), plus 1% additional removals (euthanasia or perioperative death), distributed across rural and urban scenarios.	Derived from national survey of animal welfare organizations (2024); includes humane euthanasia and surgical mortality based on expert estimates; proportional allocation by scenario.

**Table 3:** Aggregate Population Evolution under the Three Simulated Scenarios. Source: VORTEX v10.6 simulations based on DGDA data (2023–2025) and PACF parameters [22].

Scenario	Annual Sterilization Rate	Year of Saturation (K)	Demographic Trend (Year 20)	Total Population (Year 20)	Variation vs. 2025	Control Effectiveness
A (4%)	Very low	Years 4–5	Constant saturation (K)	242,940	+296%	None
B (20%)	Low	Years 4–5	Constant saturation (K)	242,940	+294%	None
C (PACF 60–70%)	High	Does not reach K	Sustained reduction	27,101	–55.9%	High

## Data Availability

The original contributions presented in this study are included in the article. Further inquiries can be directed to the corresponding author.
